# Mercury Content in Dietary Supplements From Poland Containing Ingredients of Plant Origin: A Safety Assessment

**DOI:** 10.3389/fphar.2021.738549

**Published:** 2021-11-03

**Authors:** Anna Puścion-Jakubik, Anita Mielech, Dominika Abramiuk, Małgorzata Iwaniuk, Monika Grabia, Joanna Bielecka, Renata Markiewicz-Żukowska, Katarzyna Socha

**Affiliations:** Department of Bromatology, Faculty of Pharmacy with the Division of Laboratory Medicine, Medical University of Białystok, Białystok, Poland

**Keywords:** mercury, dietary supplements, herbs, food safety, PTWI

## Abstract

Mercury (Hg) is a fairly common environmental pollutant. Chronic exposure to this element may cause, inter alia, kidney damage, and disturbances in the functioning of the nervous system. Literature data indicate that food, including dietary supplements (DS), may sometimes be contaminated with Hg. Therefore, the aim of the study was to assess Hg content in DS containing ingredients of plant origin. The study covered 200 DS available for sale in Poland. Hg content was determined by using the AAS method with the amalgamation technique using the AMA-254 analyzer. The highest average Hg content was found in preparations used as adjuncts for lowering glucose levels (23.97 ± 38.56 μg/kg). The highest percentage of PTWI (1.143%) was found in DS aimed at improving vitality. Due to the fact that DS are commonly used, their quality should be constantly monitored.

## Introduction

Dietary supplements (DS) constitute a large group of food products. They are used by patients for prophylactic purposes, to support therapy or to supplement the diet with missing nutrients, and are sometimes treated as drugs (due to their similarity in terms of pharmaceutical form). It is also commonly believed that DS have a natural composition, are not harmful, have no side effects, and cannot be overdosed. Since they are sold for example in the form of tablets, capsules, syrups, and lozenges, they tend to be mistaken for medical products. As a result, they are often used by people with weakened immunity, diseases of various systems, or confirmed vitamin and mineral deficiencies. Data show that the popularity of DS and their presence in the market are steadily increasing (The Law on Food and Nutrition Safety, 2006; Main Sanitary Inspectorate, 2021).

Among DS available in Poland, the largest market share is held by preparations with magnesium (7.56%); immunostimulants (6.58%); probiotics (6.13%); supplements for strengthening bones, muscles, and joints (4.75%); vitamins and minerals for adults (4.65%); beauty supplements (4.40%); and food supplements with substances which improve vision (4.08%) (Dietary supplements, 2017).

The danger of using DS is related to the registration procedures, which are extremely straightforward. DS are not subjected to detailed quantitative or qualitative tests, confirming their high quality and safety. Unlike in the case of drugs, during the registration process, it is not necessary to prove that a supplement actually contains the ingredients that it claims to contain or present the results of research on their safety (Regulation of the Minister of Health, 2011; Main Sanitary Inspectorate, 2021).

Mercury (Hg) is a major food contaminant. This toxic element can be released from primary natural sources (e.g., volcanic activity), primary anthropogenic sources (e.g., mining or natural gas extraction), or secondary anthropogenic sources (industrial processes). It is widely distributed in the environment; therefore, the general population is unable to avoid exposure to it (Vianna et al., 2019). Hg occurs mainly in three forms: elemental, inorganic (e.g., as mercury (I) chloride, mercury (II) chloride, or mercury (II) sulfide), and organic (methylmercury, dimethylmercury, ethylmercury, or phenylmercury) (EPoCitF, 2012). The abovementioned compounds are characterized by different bioavailability and toxic effects. The latter group includes methylmercury, which is the most common form of Hg in the food chain. For most people, the main source of exposure is diet, particularly one rich in fish and seafood (Communication from the Co, 2005; Richardson et al., 2011).

Chronic exposure to Hg can result in a number of health consequences, including disorders of the nervous system and kidneys (Johnson-Arbor et al., 2021; Novo et al., 2021). Metallic Hg enters the body *via* inhalation. Inorganic Hg compounds can mainly result in gastrointestinal disturbances and damage to the renal tubules, as well as the formation of free radicals that destroy DNA. Among the organic forms, methylmercury is most dangerous because of its high toxicity to humans. Approximately 95% of it is absorbed from food. Erythrocytes are the main accumulation site of methylmercury. Unfortunately, Hg can also penetrate the placenta and fetus and even cross the blood–brain barrier and the blood–cerebrospinal fluid barrier. Other health consequences include muscle weakness, peripheral vision disorders, problems with coordination of movements, and speech, hearing, and walking impairment (Bernhoft, 2012).

Contamination with this element has been highlighted in many reports (Kabata-Pendias, 2007; CD, 2008; Rice et al., 2014). However, it should be emphasized that there is low public awareness of the health consequences of excessive consumption of DS. Traditional herbal remedies from Asia (EPoCitF, 2012) may pose the greatest threat. It was shown that 17% of traditional herbal preparations exceeded the safety limits for Hg (Martena et al., 2010).

In 2008, the European Union set (Commission Regulation, 2008) the maximum permissible level of Hg in DS at 0.1 mg/kg. However, the European Commission Regulation (Commission Regulation, 2018) does not include data on Hg content in DS, which may indicate that this source presents a lower health risk than other food products listed in the regulation, for example, tree nuts (standard: 0.02 mg/kg), edible herbs and flowers (0.03 mg/kg), wild mushrooms (0.5 mg/kg), oilseeds (0.02 mg/kg), teas, coffee beans, or herbal infusions (0.02 mg/kg).

Our previous studies of 30 plant-based DS revealed contamination with Hg. The highest average content was found in DS supporting immunity (9.62–17.1 μg/kg) and those for the urinary system (9.98–21.2 μg/kg) (Socha et al., 2013). Alarming data were published in 2018. Hg content of 4212.04 μg/kg and 1806.12 μg/kg was detected in DS containing the following ingredients of plant origin: bamboo shoots (85.72 mg/portion), horsetail (52.63 mg/portion), and algae *Chlorella pyrenoidosa* Chick (100% of algae, no specific data on the content) (Brodziak-Dopierała et al., 2018). The abovementioned data indicate that supplements containing herbs may still be a cause for concern and require strict control.

Therefore, the aim of our research was to evaluate the content of Hg in DS containing ingredients of plant origin. In addition, exposure indicators related to the regular use of Hg-contaminated DS were assessed, which made it possible to perform such a comprehensive assessment of exposure to the abovementioned element.

## Materials and Methods

### Materials

In this research, 200 DS were included. All the analyzed products were available for sale in Poland. The study samples consisted of DS for the following: acne therapy support (*n* = 6), cholesterol control (*n* = 7), detoxification (*n* = 5), digestive tract support (*n* = 21), glucose level control (*n* = 5), for hair, skin and nails, called nutricosmetics (*n* = 17), immunity (*n* = 18), memory (*n* = 9), the nervous system (*n* = 4), sore throat (*n* = 11), the urinary tract (*n* = 10), for veins (*n* = 6), for vision and eye health (*n* = 5), vitality (*n* = 17), and supplements containing vitamins and minerals (*n* = 23) for weight loss (*n* = 25) and others (*n* = 11). All the studied DS contained ingredients of plant origin.

The DS were purchased in stationery and online drugstores belonging to nationwide pharmacy chains.

### Methods

#### Preparation of DS for Analysis

Solid DS were homogenized in a vibrating mill (Testchem, Radlin, Poland), while liquid ones were mixed using a Vortex Mixer Benchmixer (Benchmark, Sayreville, NY, United States of America). The weighed samples (0.02 g or 50 μL, with an accuracy of 1 mg) were placed in a cuvette, and Hg content was determined.

#### Determination of Hg Content

The content of Hg was measured using atomic absorption spectrometry (AAS), using an Advanced Mercury Analizer (AMA)-254 (Leco Corp. Altec Ltd. Prague, Czech Republic), according to the methodology described previously (Bielecka et al., 2020). This method facilitates the separation of Hg from its compounds, both inorganic and organic, and transforming it into an atomic form.

The process of determining the content of Hg consisted of 3 steps. The first step was to dry the sample and then burn it in an oxygen stream; medicinal oxygen was used as the carrier gas. The second step was to pass the released Hg vapor through the catalytic column; the vapors were captured by the amalgamator. The third step was to release Hg from the amalgamator and measure its content using the AAS method at a wavelength of 254 nm. The method’s limit of quantification was 0.003 ng Hg/g sample.

#### Quality Control of the Method

Quality control of the method was performed using certified reference material – *Mixed Polish Herbs* (INCT-MPH-2), obtained from the Institute of Nuclear Chemistry and Technology (Warsaw, Poland). The particular analytical steps were analogous to the procedure for determining Hg content in the samples. The recovery rate was 102%, and the precision rate was 2.1%.

#### Comparison to the Norm

The obtained results were compared to the applicable Commission Regulation (Commission Regulation, 2008), establishing the maximum levels of certain contaminants in foodstuffs, according to which the maximum level of Hg in DS is 0.1 mg/kg.

#### Assessment of Consumption Safety

The risk of the health consequences related to the consumption of Hg in DS was estimated by calculating selected exposure indicators, such as the estimated daily intake (EDI), the estimated weekly intake (EWI), the percentage of provisional tolerable weekly intake (% PTWI), and Hg consumption during 1 month and 1 year. The EDI [µg/day] was calculated using the following formula:

EDI =C ×Cons, 

where C [µg/kg] is the concentration of Hg in the sample and Cons [kg] is the daily portion of the studied supplement, considering the weight of the portion and maximum daily dosage. The EWI [µg/week] was estimated by multiplying the EDI value by seven (which corresponds to 1 week). To determine the % PTWI [µg/kg/week], the following equation was used:

$$\%PTWI\;=\left[\left(\frac{EWI}{BW}\right)/4\right]*100,$$

where BW is the average body weight of an adult in Poland (the weight of 70 kg was assumed). The obtained results were compared to the norm established by the European Food Safety Authority at 4 µg/BW/week (EPoCitF, 2012).

Moreover, the THQ index (target hazard quotient) was calculated for selected DS using the following formula:

$$THQ=\frac{Fr\;x\;D\;x\;Cons\;x\;C}{RfD\;x\;BW\;x\;T}\;x\;10^{-3},$$

where Fr is the frequency of exposure [365 days/year], D is the time of exposure [70 years], Cons is the average DS consumption per day [g], C is the concentration of Hg in the DS [mg/kg], RfD is the oral reference dose [0.3 μg/kg body weight/day], BW is the body weight [kg], and T is the time of exposure [365 days/year x 70 years].

The interpretation of the THQ is as follows: if the THQ value is above 1, it may indicate a potential risk associated with the consumption of the heavy metal in question with the DS. On the other hand, when the value is below 1, it indicates a low non-carcinogenic risk.

The supplementation materials provide Hg content per portion (i.e., one capsule, one tablet, *etc*.), daily consumption (content in one portion multiplied by the number of portions recommended for consumption by the manufacturer), and weekly consumption – calculated analogously by multiplying the daily consumption for 7, monthly – for 30, and yearly – for 365.

#### Statistical Analysis of the Results

Data were analyzed using Statistica 13.3 (TIBCO Software Inc. Palo Alto, CA, United States). In order to assess the consistency of the data distribution with normal distribution, the Shapiro–Wilk, Kolmogorov–Smirnov, and Lilliefors tests were used. Due to the lack of normality in the data distribution, the Kruskal–Wallis analysis of variance (ANOVA) was performed to compare the content of Hg in individual categories and between pharmaceutical forms. The table lists the mean (X), standard deviation (SD), minimum (Min), and maximum (Max) levels to compare the results with the literature data, the median (Me), lower (Q1), and upper (Q3) quartile levels due to lack of normality of the data distribution. The level of statistical significance was set at *p* < 0.05.

## Results

Taking into account all studied DS (*n* = 200), the mean Hg content was 3.37 ± 7.65 μg/kg and the median content was 1.69 μg/kg, while the range of quartiles ranged from 1.10 to 2.86 μg/kg.

Table 1 shows the content of Hg in the tested DS, with division into categories. Detailed data on Hg content in individual DS are included in the Supplementary Materials section: Supplementary Tables S1–S17.

**TABLE 1:** Hg content in DS and health risks of their use.

Category of the supplements	n	Content of Hg [µg/kg]		Indicators		Intake of Hg [µg] min–max		PTWI min–max [%]
		X ± SD min-max	Me Q_1_-Q_3_	EDI [µg]	EWI [µg]	Monthly	Annual	
Acne	6	2.24 **±** 1.95	1.83	0.001–0.003	0.004–0.021	0.015–0.088	0.186–1.069	0.001–0.007
		0.71–6.01	1.06–2.17					
Cholesterol control	7	1.29 **±** 0.56	1.32	0.001–0.002	0.004–0.012	0.016–0.051	0.192–0.616	0.001–0.004
		0.42–2.16	1.00–1.55					
Detox	5	1.20 **±** 1.30	0.64	0.001–0.017	0.004–0.117	0.017–0.502	0.206–6.109	0.001–0.042
		0.49–3.52	0.65–0.68					
Digestive tract	21	1.91 **±** 1.66	1.42	0.000–0.014	0.001–0.097	0.002–0.418	0.029–5.082	<0.001–0.035
		0.23–7.10	0.91–2.14					
Glucose level	5	23.97 **±** 38.56	5.94	0.001–0.119	0.004–0.830	0.018–3.558	0.223–43.283	0.002–0.296
		0.80–91.40	0.99–20.72					
Nutricosmetics	17	4.13 **±** 5.32	2.48	0.000–0.068	0.003–0.474	0.013–2.030	0.162–24.693	0.001–0.169
		0.56–22.00	1.20–3.72					
Immunity	18	2.62 **±** 2.73	1.90	0.001–0.047	0.005–0.327	0.022–1.399	0.263–17.025	0.002–0.117
		0.34–11.88	0.90–2.71					
Memory	9	1.45 **±** 0.98	1.46	0.000–0.006	0.003–0.041	0.012–0.177	0.147–2.159	0.001–0.015
		0.66–3.79	0.92–2.69					
Nervous system	4	2.41 **±** 1.64	2.09	0.002–0.009	0.011–0.065	0.046–0.277	0.564–3.371	0.004–0.023
		0.80–4.64	1.49–3.01					
Throat	11	1.54 **±** 0.87	1.50	0.003–0.050	0.018–0.352	0.078–1.510	0.948–18.371	0.006–0.126
		0.27–3.20	1.12–1.99					
Urinary tract	10	4.49 **±** 4.90	1.99	0.001–0.012	0.005–0.082	0.019–0.350	0.236–4.264	0.002–0.029
		0.67–13.84	1.37–7.84					
Veins	6	4.50 **±** 2.49	3.76	0.001–0.011	0.005–0.076	0.021–0.324	0.252–3.947	0.002–0.027
		1.27–8.57	3.66–5.44					
Vision	5	6.06 **±** 8.91	1.39	0.001–0.018	0.004–0.126	0.016–0.538	0.196–6.544	0.001–0.045
		1.27–21.81	1.38–4.46					
Vitality	17	2.06 **±** 1.38	1.81	0.000–0.457	0.003–3.201	0.014–13.720	0.172–166.932	0.001–1.143
		0.46–5.32	1.11–2.65					
Vitamins and minerals	23	4.21 **±** 8.61	1.86	0.001–0.036	0.006–0.252	0.026–1.079	0.312–13.124	0.002–0.090
		0.57–42.96	1.18–3.35					
Weight loss	25	3.08 **±** 3.62	1.78	0.001–0.026	0.004–0.184	0.018–0.790	0.215–9.617	0.001–0.066
		0.52–17.26	1.21–2.88					
Other	11	1.56 **±** 0.69	1.36	0.000–0.023	0.001–0.163	0.006–0.699	0.070–8.509	<0.001–0.058
		0.54–2.47	1.09–2.29					

X–average, SD–standard deviation, Me–median, Q1–quartile 1, Q3–quartile 3, EDI–estimated daily intake, EWI–estimated weekly intake, PTWI–provisional tolerable weekly intake.

In this study, the highest median Hg content (5.94 μg/kg) was detected in the group of supplements responsible for controlling glucose levels, while the lowest was in the detoxifying supplements (0.64 μg/kg). The average concentrations of Hg ranged from 0.23 μg/kg in a supplement for the digestive tract to 91.40 μg/kg in a product designed to control blood glucose levels.

Among the supplements supporting acne treatment, the highest content of Hg (6.01 μg/kg) was found in a supplement containing the extract of *Viola tricolor* L. (wild pansy) (100 mg).

In the case of DS used to help in reducing cholesterol levels, the highest content of Hg (2.16 μg/kg) was found in a supplement containing red yeast rice extract with monacolin K (250 mg) and phytosterols (47.5 mg).

Considering the subgroup comprising detoxifying supplements (n = 5), the maximum Hg concentration (3.52 μg/kg) was detected in products containing silver birch extract (600 mg), common dandelion extract (350 mg), *Orthosiphon spicatus (*Thunb.*)* Backer, Bakh. f. Steenis not Benth. extract (350 mg), ginseng root extract (300 mg), white nettle extract (300 mg), broadleaf plantain extract (250 mg), *Pilosella officinarum* Vaill. extract (250 mg), maypop extract (200 mg), fennel extract (175 mg), olive extract (175 mg), green tea extract (100 mg), and lemon extract (100 mg).

Among the DS recommended to improve digestive tract function (*n* = 21), the greatest amount of Hg (7.10 μg/kg) was detected in a sample containing mint leaves.

The next studied subgroup included supplements for glucose level control (*n* = 5). In this subgroup, one sample with the highest Hg concentration, among whose ingredients were extracts of *Gymnema sylvestre* R. Br. and *Trigonella foenum graceum* L. (fenugreek), contained the highest level of detected Hg, which was nearly the maximum permissible amount of Hg (91.40 μg/kg).

In the subgroup of supplements with bioactive substances, which could play a role in improving the condition of hair, skin, and nails (*n* = 17), the highest level of Hg (22.00 μg/kg) was observed in a sample containing *Chlorella pyrenoidosa* Chick (200 mg).

Analyses of Hg content in the subgroup of supplements recommended to strengthen the immune system (n = 18) revealed the highest amount of the element (11.88 μg/kg) in a sample containing the extract of acerola.

Among the DS recommended for memory support (*n* = 9), the highest level of Hg (3.79 μg/kg) was recorded in supplements with extracts of *Panax ginseng* C.A. Meyer (71.43 mg), *Ilex paraguariensis* A. St.-Hill (150 mg), and *Bacopa monnieri* (L.) Wettst (36 mg).

In the case of supplements supporting the functioning of the nervous system (n = 4), the greatest Hg level (4.64 μg/kg) was found in a sample containing lemon balm leaf.

In our study, the highest Hg (3.20 μg/kg) concentration in the analyzed DS for patients with throat symptoms (*n* = 11) was observed in products containing extracts of *Salvia officinalis* L. (11.25 mg), *Althaea officinalis* L. (11.25 mg), *Tilia cordata* Mill (10 mg), *Matricaria recutita* L. (8 mg), propolis (5.25 mg), *Sambucus nigra* L. (3.75 mg), and *Thymus vulgaris* L. (6.25 mg).

Ten of the tested products were dedicated to supporting the urinary tract. In this subgroup, the highest content of Hg (13.84 μg/kg) was detected in DS based on cranberry fruit extract (360 mg).

The maximum Hg content in the next studied group, that is, supplements to improve the condition of veins (*n* = 6) was 8.57 μg/kg. These DS were based on *Vitis vinifera* L. leaf extract (84 mg), and grape seed extract (79 mg).

In the subgroup of supplements claiming to support vision (*n* = 5), the highest level of Hg (21.81 μg/kg) was found in supplements containing bilberry fruit extract (290 mg) and Aztec marigold flower extract (15 mg).

Among the DS recommended to improve vitality (*n* = 17), the highest content of the tested toxic element (5.32 μg/kg) was found in a sample based on *Ginseng* extract C.A. Meyer (50 mg) and ginkgo extract (40 mg).

In the next studied subgroup—DS containing vitamins and minerals—the highest concentration of Hg (42.96 μg/kg) was detected in a supplement containing extract of *Withania somnifera* (L.) Dunal (80 mg).

The largest surveyed group (*n* = 25) included products designed to promote weight loss. The highest concentration of Hg (17.26 μg/kg) was observed in one sample containing the following substances: extract of *Camellia sinensis* (L.) Kuntze (105 mg), extract of *Zingiber officinale* Rosc (100 mg), extract of cayenne peper (70 mg), extract of green coffee (5 mg), extract of *Cinnamomum* Scheffer (5 mg), extract of *Paullinia cupana* Kunth (6.6 mg), extract of *Citrus sinensis* (L.) Osbeck, extract of *Citrus grandis* Osbeck, and extract of *Citrus aurantium vel. dulcis* L. (10 mg).

In our research, 11 products were not classified into any of the studied subgroups. The highest Hg content (2.47 μg/kg) was found in a product containing extracts of *Melissa officinalis* L. leaves (300 mg), *Humulus lupulus* L. (100 mg), and *Rhodiola rosea* L. (100 mg).

**FIGURE 1:**
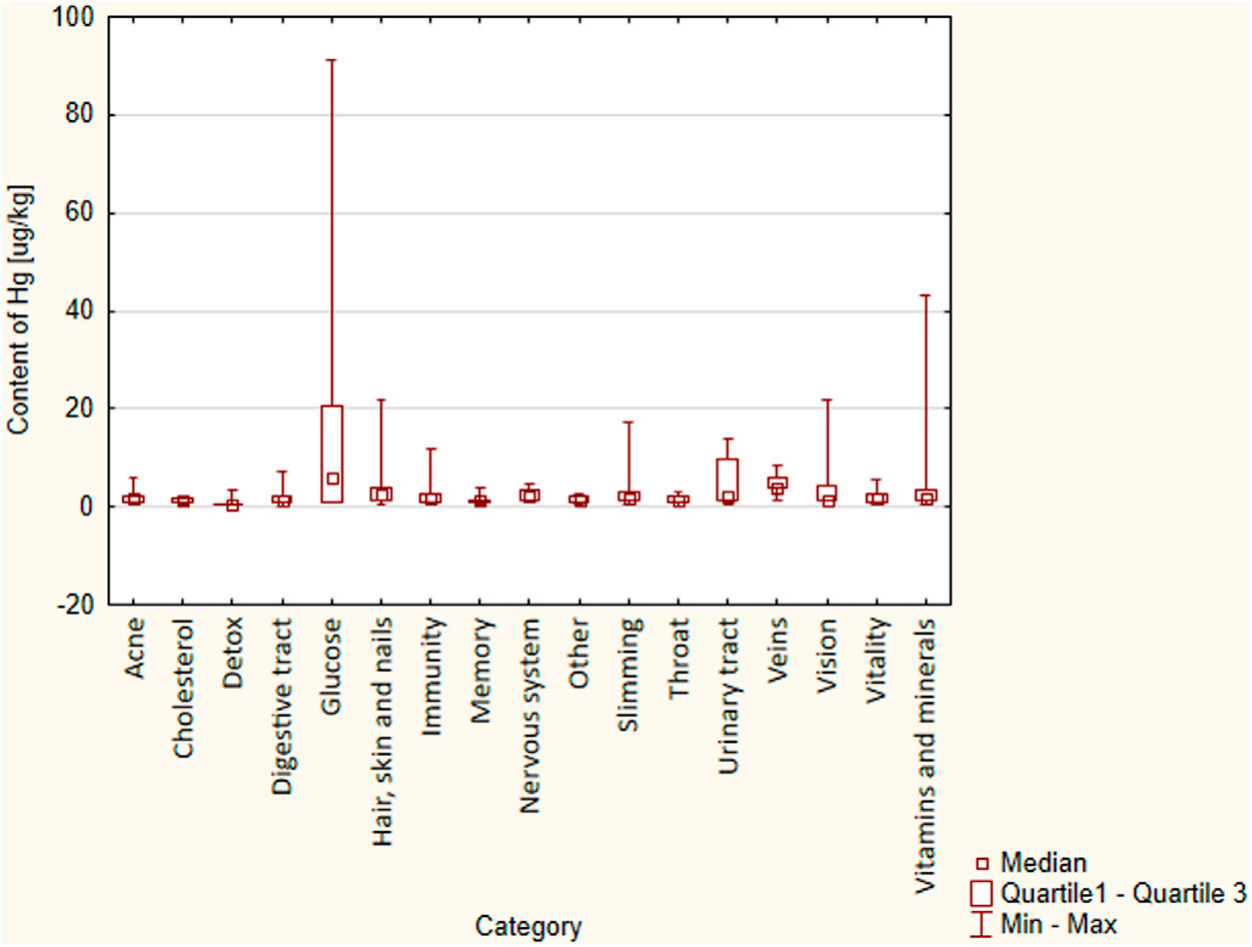
Differences in Hg content between the category of DS (*p* = 0.068, results were not statistically significant).

The study showed that the examined categories of DS did not differ significantly in terms of Hg content (*p* = 0.068) (Figure 1).

**FIGURE 2:**
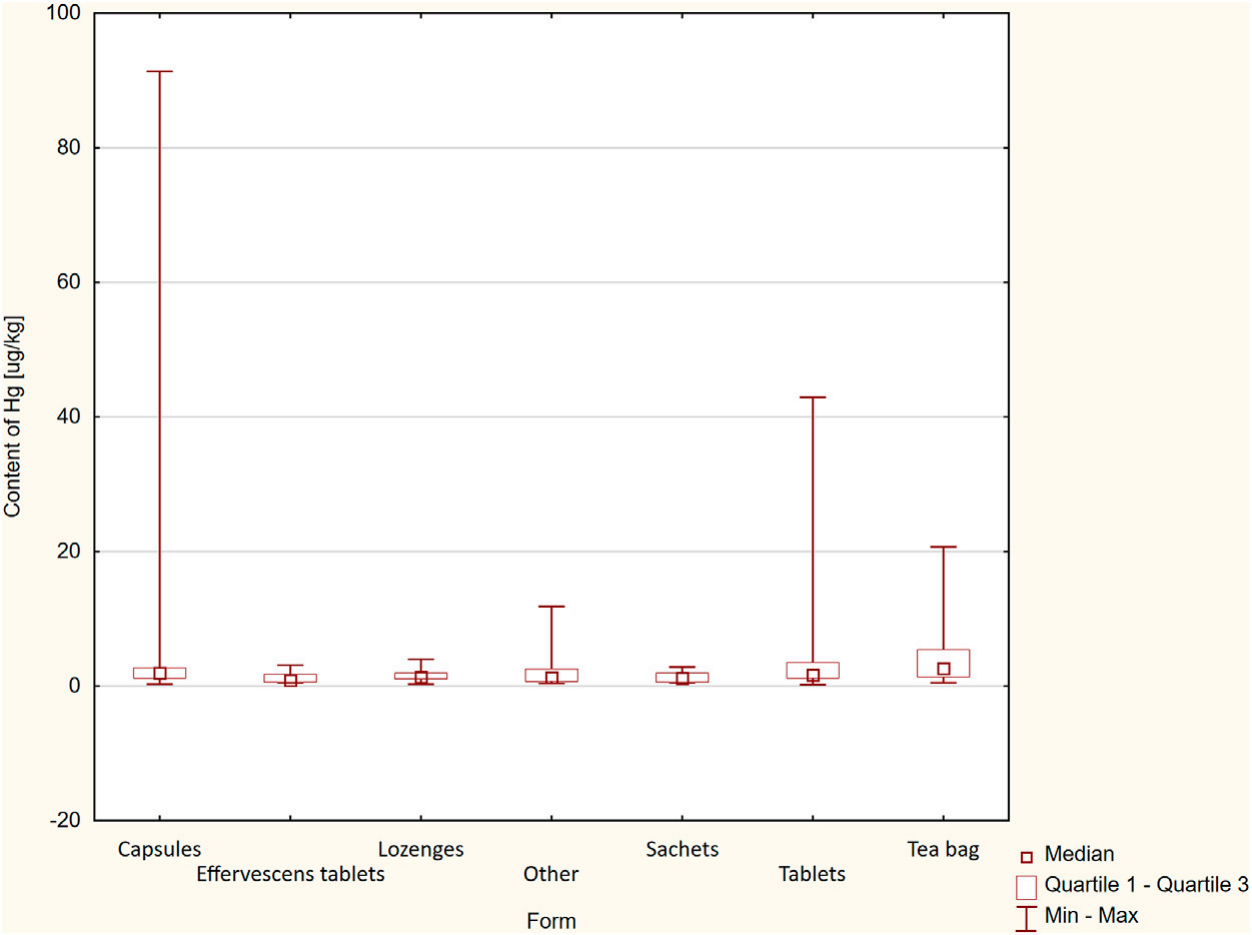
Differences in Hg content between forms of DS (*p* = 0.045).

Moreover, it was assessed that the pharmaceutical form of the DS affected the content of the tested element–*p* = 0.045 (Figure 2). The preparations available in the form of sachets for infusion were characterized by the highest median: 2.66 μg/kg (Q1-Q3: 1.36–5.49 μg/kg).

Our analyses showed that the content of Hg in all tested DS was below 0.1 mg/kg. In the case of one DS, the content of this element was above 0.09 mg/kg, which is a value close to the maximum allowable concentration.

The calculated percentage of the PTWI for Hg due to intake of studied DS is included in Table 1. The lowest percentage of the PTWI was lower than 0.001%, while the highest percentage of the PTWI was calculated for one DS designed to boost vitality (1.143%) (Table S14). Generally, the values of this indicator in the vast majority of samples were lower than 1%, ranging from 0.001 to 0.030%.

In the case of the DS with the highest content per sample (category: glucose, 91.40 μg/kg) and the one with which the most Hg would be consumed during a single day (category: vitality, 22.00 μg/kg, it is recommended to consume 15 servings per day), the THQ index was calculated as follows: 4.10E-03 and 3.22E-03, respectively.

## Discussion

DS are used by consumers from different age groups. Due to their similarity to medicinal products, they are applied in the treatment of various diseases. For the abovementioned reason, they should be of high quality. Registration procedures and national regulations do not require qualitative research; therefore, this kind of research is of interest to various authors.

Our research has shown that the highest average Hg content was found in DS used for lowering glucose levels (23.97 ± 38.56 μg/kg). The supplement with the highest concentration of the element (91.40 μg/kg) contained two ingredients of plant origin, namely, extract of *Gymnema sylvestre* R. Br (185 mg) and extract of *Trigonella foenum graceum* L./fenugreek (90 mg). Moreover, they contain chromium and lipoic acid as well.

It can be assumed that high concentration of Hg in *Gymnea sylvestrae* R. Br. may stem from the fact that the plant is mainly grown in Asia. China is a country where Hg is the most prevalent toxic element (Huang et al., 2022); hence, plants are likely to absorb it.

To our knowledge, the research conducted in this project involved more DS than in most previous publications to better assess consumer exposure. Research on Hg content in 24 DS containing ingredients of plant origin was carried out by Brodziak–Dopierała et al. (Brodziak-Dopierała et al., 2018). The authors measured the content of Hg using the same method that we did and found that the studied supplements contained 0.02 to 4,293.07 μg/kg of Hg. The average concentration was almost 58 times higher than that shown in our study (193.77 vs 3.37 μg/kg). The second highest result (1806.12 μg/kg) involved a DS containing *Chlorella pyrenoidosa* Chick algae. In our study, a *Chlorella*-based product with the recommended dose of as many as 15 tablets/day also proved to have one of the highest amounts of Hg (22.001 μg/kg). The result obtained by us, however, was as much as 82 times lower than the highest one in the abovementioned studies. Moreover, the authors showed that the preparations in tablets were characterized by a significantly higher mean Hg content compared to capsules (274.80 ± 917.64 μg/kg vs 5.95 ± 7.30 μg/kg). Our research looked at more pharmaceutical forms and revealed that the infusion bags had the highest median Hg content.

*Chlorella* has good Hg absorption properties. It prevents the reabsorption of Hg from the gastrointestinal tract; therefore, it can be used as an effective absorbent to remove Hg from the body (Yadav et al., 2020). In a study conducted by Caldas et al., Hg was detected in all samples, while none of the samples exceeded the acceptable limit (from <0.01 to 0.09 μg/g) (Caldas and Machado, 2004). On the other hand, in a study analyzing the Polish supplement market, a preparation based on *Chlorella* had one of the highest concentrations of Hg: 1810 μg/kg, which exceeded the acceptable standard (100 μg/kg) (Brodziak-Dopierała et al., 2018).

Other data, including Hg content in 24 DS, come from Mexico. The content of the discussed element ranged below 240–850 μg/kg. According to the authors, Hg at the detection limit level was present in only 5 DS (about 21% of samples). The highest content (850 μg/kg) was found in a DS containing Guaco stem (*Mikania guaco* Bonpl), red vine leaves (*Vitis viniferol* L.), horse plant (*Equisetum arvense* L.), and gorongoro bark (García-Rico et al., 2007). Hg content in DS can be explained by the presence of this ingredient in the form of cinnabar (HgS), especially in Chinese preparations, including those used for the treatment of throat diseases (Bin et al., 2001; Wu et al., 2002).

Studies assessing the quality of 49 pharmaceutical products from Korea containing raw materials of plant origin showed that the content of total Hg in these preparations was high. For example, the highest amount was found in a preparation containing royal jelly—as much as 159.89 μg/kg—which is about 1.76 times higher than the highest result obtained by us. The mean methylmercury content of the herbal preparations in this study was 31.18 μg/kg, while the preparations containing Spirulina had 0.62 μg/kg of Hg (Lee and Lee, 2013).

Another study of DS from Poland assessed the quality of 33 products containing macro and microelements (*n* = 7), vitamins (*n* = 5), and nutricosmetics (*n* = 6) and classified as “other” (*n* = 15). The average content of Hg was 5.5 μg/kg, with the highest in a preparation containing vitamin C and rutin (16.7 μg/kg) (Kowalski and Frankowski, 2015).

Another study of DS from Poland involved a quality assessment of 41 DS containing terrestrial plants and microalgae. The authors showed that 29.3% of the investigated DS were contaminated with Hg. The average content of Hg in the products containing ingredients of plant origin was 5 ± 8 μg/kg, while in those based on microalgae — 3 ± 6 μg/kg. The highest concentration (28 μg/kg) was found in tablets which contained *Rehnabbua glutinosa* (Gaertn.) Steud. radix and Wolfiporia (Ćwieląg-Drabek et al., 2020).

The prevalence of Hg contamination in Ayurvedic herbal DS was demonstrated by Mikulski et al. (Mikulski et al., 2017). The presence of Hg was detected in as many as 38% of the tested preparations. It is very disturbing that the content of the toxic element ranged from 800 to 279, 000, 000 μg/kg. Brihat Vatchintamani Ras (139,500 µg/0.5 g pill) was characterized by the highest content of Hg per one pill.

In contrast, studies of 10 DS from Turkey, carried out using the ICP-OES method, showed no Hg in the tested preparations (Canbay and Doğantűrk, 2017).

The presence of Hg in DS containing ingredients of plant origin can be explained by the fact that plants are one of the best agents for removing Hg2 + impurities from soil. Bioabsorption is based on mechanisms such as chelation, ion exchange, and species of the structural polysaccharide cell wall network absorption by physical forces and ion entrapment in inter- and intra-fibrillar capillaries. For example, Hg is selectively accumulated by *Carica papaya* L. wood or *Ricinus communis* L. (Basha et al., 2009; Kumar et al., 2017). It should be emphasized that the absorption of Hg may be toxic not only to humans but also to plants themselves.

DS can be one of the sources of exposure to Hg. Other sources of exposure to organic forms of Hg include fossil fuel emissions, medical waste incineration, dental amalgams, and various other products, including skin creams, bactericidal soaps, teething powders, painkillers, thermometers, blood pressure gauges, barometers, bulbs, and batteries. Other sources of organic Hg include phenylmercury and ethylmercury compounds, which used to be components of latex paints before the 1990s, and thimerosal, which was used as a preservative in vaccines (Rice et al., 2014).

Chronic exposure to Hg may result in, inter alia, disruption of the endocrine system. Hg is mainly stored in the thyroid and pituitary gland. Previous research has shown that the concentration of the element in these organs ranged from 6.3 to 77 ng/g, while in another study, it amounted to 28 ng/g. These levels exert neurotoxic and cytotoxic effects (Rice et al., 2014). Exposure to Hg can cause changes in the nervous system, which is associated with a toxic increase in reactive oxygen species (Fernandes Azevedo et al., 2017). However, the THQ index calculated by us does not indicate an increased non-carcinogenic risk resulting from consuming the DS under investigation.

In another study, the DS which had the highest % PTWI (3.91%) contained two ingredients of plant origin: extract of millet (50 mg) and extract of wheat germ (50 mg) (Brodziak-Dopierała et al., 2018). The highest % PTWI calculated in our study amounted to 1.143% and was detected in a preparation containing *Guarana* Kunth seed extract.

Hg, along with several other elements (Cd and Pb), has been recognized as an impurity arising from the food chain (Tchounwou et al., 2012), which is also confirmed by our study. Among the many factors affecting the concentration of Hg in food are natural factors, including not only growing conditions (type of water and soil) and cultivation practices but also meteorological conditions (i.e. geological areas for Hg-rich formations and atmospheric deposition rate). The fact that supplement ingredients are often plant-based may account for Hg contamination. Plants quite easily absorb heavy metals from soil and water, and these can remain in the final product, even after processing. It should also be emphasized that the location of the source of raw materials for the production of supplements is of considerable importance and may directly affect the content of Hg in the final product (Bandara et al., 2020).

Summing up, although the content of Hg in the studied DS was lower than that reported in most of the literature and did not exceed the permissible maximum content prescribed by law, it should be emphasized that since Hg is a toxic element, any amount of it may be harmful to health. During the production of DS, strict procedures for obtaining raw materials from crops controlled in terms of environmental pollution, including soil, as well as procedures for cleaning plant materials and eliminating the risk of contamination of the final product at all stages should be implemented.

## Conclusion

DS containing ingredients of plant origin are mostly safe in terms of Hg content, but it should be stressed that Hg is a highly toxic element, and its long-term use may pose a health hazard. This is especially dangerous in the case of chronically ill people who use several DS at the same time. Consumers and pharmacists should pay attention to the origin of DS and the recommended number of tablets taken during the day, as in some cases, higher doses can lead to increased exposure to Hg. In addition, there is a recognized need for DS to be tested for quality and safety before being placed on the market.

## Data Availability

The results of the research carried out may be available from the authors. Requests to access the datasets should be directed to anna.puscion-jakubik@umb.edu.pl.
