# Supplementary material for: Mercury Content in Dietary Supplements From Poland Containing Ingredients of Plant Origin: A Safety Assessment
Source: Front Pharmacol. 2021 Nov 3;12:738549. doi: 10.3389/fphar.2021.738549 (PMC8595131; doi:10.3389/fphar.2021.738549)
Supplement: Supplementary file 1 [file DataSheet1.docx]

Table S1. Mercury content in dietary supplements supporting acne therapy.

| **No** | **Max**  **daily dosage** | **Content of Hg [µg/kg]** | **Weight of 1 portion**  **[g]** | **Intake of Hg [µg]** | | | | | **% PTWI** |
| --- | --- | --- | --- | --- | --- | --- | --- | --- | --- |
|  |  |  |  | **One portion** | **Daily (EDI)** | **Weekly (EWI)** | **Monthly** | **Annual** |  |
| 1. | 2 | 0.71 | 0.868 | 0.00062 | 0.00123 | 0.00862 | 0.03692 | 0.44925 | 0.003 |
| 2. | 1 | 1.77 | 0.540 | 0.00096 | 0.00096 | 0.00669 | 0.02869 | 0.34906 | 0.002 |
| 3. | 1 | 0.82 | 0.625 | 0.00051 | 0.00051 | 0.00357 | 0.01532 | 0.18638 | 0.001 |
| 4. | 1 | 1.88 | 0.619 | 0.00116 | 0.00116 | 0.00815 | 0.03491 | 0.42476 | 0.003 |
| 5. | 1 | 6.01 | 0.487 | 0.00293 | 0.00293 | 0.02050 | 0.08786 | 1.06902 | 0.007 |
| 6. | 1 | 2.26 | 0.481 | 0.00109 | 0.00109 | 0.00761 | 0.03263 | 0.39695 | 0.003 |

Table S2. Mercury content in dietary supplements for the cholesterol control.

| **No** | **Max**  **daily dosage** | **Content of Hg [µg/kg]** | **Weight of 1 portion [g]** | **Intake of Hg [µg]** | | | | | **% PTWI** |
| --- | --- | --- | --- | --- | --- | --- | --- | --- | --- |
|  |  |  |  | **One portion** | **Daily (EDI)** | **Weekly (EWI)** | **Monthly** | **Annual** |  |
| 1. | 1 | 1.10 | 0.752 | 0.00083 | 0.00083 | 0.00581 | 0.02488 | 0.30275 | 0.002 |
| 2. | 1 | 1.32 | 0.738 | 0.00097 | 0.00097 | 0.00682 | 0.02925 | 0.35584 | 0.002 |
| 3. | 1 | 2.16 | 0.403 | 0.00087 | 0.00087 | 0.00610 | 0.02615 | 0.31817 | 0.002 |
| 4. | 1 | 1.66 | 0.478 | 0.00079 | 0.00079 | 0.00554 | 0.02376 | 0.28910 | 0.002 |
| 5. | 2 | 1.45 | 0.584 | 0.00084 | 0.00169 | 0.01182 | 0.05067 | 0.61646 | 0.004 |
| 6. | 1 | 0.90 | 0.792 | 0.00071 | 0.00071 | 0.00500 | 0.02143 | 0.26075 | 0.002 |
| 7. | 2 | 0.42 | 0.621 | 0.00026 | 0.00053 | 0.00369 | 0.01580 | 0.19221 | 0.001 |

Table S3. Mercury content in dietary supplements for detoxifying.

| **No** | **Max**  **daily dosage** | **Content of Hg [µg/kg]** | **Weight of 1 portion [g]** | **Intake of Hg [µg]** | | | | | **% PTWI** |
| --- | --- | --- | --- | --- | --- | --- | --- | --- | --- |
|  |  |  |  | **One portion** | **Daily (EDI)** | **Weekly (EWI)** | **Monthly** | **Annual** |  |
| 1. | 1 | 0.64 | 0.888 | 0.00056 | 0.00056 | 0.00395 | 0.01692 | 0.20582 | 0.001 |
| 2. | 1 | 3.52 | 0.936 | 0.00330 | 0.00330 | 0.02307 | 0.09887 | 1.20291 | 0.008 |
| 3. | 2 | 0.68 | 0.789 | 0.00054 | 0.00108 | 0.00756 | 0.03238 | 0.39396 | 0.003 |
| 4. | 1 | 0.49 | 1.923 | 0.00094 | 0.00094 | 0.00661 | 0.02833 | 0.34463 | 0.002 |
| 5. | 1 | 0.64 | 26.028 | 0.01674 | 0.01674 | 0.11715 | 0.50208 | 6.10864 | 0.042 |

Table S4. Mercury content in dietary supplements supporting the functioning of the digestive tract.

| **No** | **Max**  **daily dosage** | **Content of Hg [µg/kg]** | **Weight of 1 portion [g]** | **Intake of Hg [µg]** | | | | | **% PTWI** |
| --- | --- | --- | --- | --- | --- | --- | --- | --- | --- |
|  |  |  |  | **One portion** | **Daily (EDI)** | **Weekly (EWI)** | **Monthly** | **Annual** |  |
| 1. | 2 | 0.52 | 4.891 | 0.00254 | 0.00508 | 0.03554 | 0.15231 | 1.85305 | 0.013 |
| 2. | 3 | 2.02 | 0.325 | 0.00066 | 0.00197 | 0.01377 | 0.05900 | 0.71780 | 0.005 |
| 3. | 3 | 2.20 | 0.405 | 0.00089 | 0.00267 | 0.01871 | 0.08019 | 0.97565 | 0.007 |
| 4. | 1 | 0.23 | 0.349 | 0.00008 | 0.00008 | 0.00056 | 0.00241 | 0.02930 | <0.001 |
| 5. | 2 | 1.67 | 0.357 | 0.00060 | 0.00119 | 0.00836 | 0.03584 | 0.43600 | 0.003 |
| 6. | 2 | 1.42 | 4.916 | 0.00696 | 0.01392 | 0.09745 | 0.41766 | 5.08157 | 0.035 |
| 7. | 2 | 1.23 | 4.903 | 0.00603 | 0.01205 | 0.08436 | 0.36155 | 4.39882 | 0.030 |
| 8. | 4 | 2.45 | 0.605 | 0.00148 | 0.00592 | 0.04145 | 0.17765 | 2.16144 | 0.015 |
| 9. | 1 | 7.10 | 1.820 | 0.01293 | 0.01293 | 0.09048 | 0.38777 | 4.71786 | 0.032 |
| 10. | 1 | 5.49 | 0.747 | 0.00410 | 0.00410 | 0.02871 | 0.12303 | 1.49688 | 0.010 |
| 11. | 1 | 3.23 | 1.436 | 0.00463 | 0.00463 | 0.03244 | 0.13902 | 1.69140 | 0.012 |
| 12. | 1 | 2.09 | 1.874 | 0.00392 | 0.00392 | 0.02744 | 0.11761 | 1.43095 | 0.010 |
| 13. | 6 | 2.14 | 0.422 | 0.00090 | 0.00543 | 0.03800 | 0.16286 | 1.98144 | 0.014 |
| 14. | 2 | 1.33 | 0.518 | 0.00069 | 0.00138 | 0.00967 | 0.04146 | 0.50444 | 0.003 |
| 15. | 6 | 1.63 | 0.457 | 0.00074 | 0.00446 | 0.03121 | 0.13375 | 1.62735 | 0.011 |
| 16. | 2 | 1.39 | 0.717 | 0.00100 | 0.00200 | 0.01397 | 0.05988 | 0.72859 | 0.005 |
| 17. | 3 | 0.52 | 0.549 | 0.00028 | 0.00085 | 0.00596 | 0.02554 | 0.31080 | 0.002 |
| 18. | 4 | 0.76 | 0.502 | 0.00038 | 0.00152 | 0.01063 | 0.04554 | 0.55409 | 0.004 |
| 19. | 2 | 0.60 | 1.059 | 0.00064 | 0.00128 | 0.00893 | 0.03825 | 0.46539 | 0.003 |
| 20. | 1 | 1.09 | 0.612 | 0.00067 | 0.00067 | 0.00467 | 0.01999 | 0.24326 | 0.002 |
| 21. | 4 | 0.91 | 1.797 | 0.00164 | 0.00656 | 0.04594 | 0.19688 | 2.39537 | 0.016 |

Table S5. Mercury content in dietary supplements for controlling the glucose levels.

| **No** | **Max**  **daily dosage** | **Content of Hg [µg/kg]** | **Weight of 1 portion [g]** | **Intake of Hg [µg]** | | | | | **% PTWI** |
| --- | --- | --- | --- | --- | --- | --- | --- | --- | --- |
|  |  |  |  | **One portion** | **Daily**  **(EDI)** | **Weekly (EWI)** | **Monthly** | **Annual** |  |
| 1. | 2 | 0.99 | 0.662 | 0.00065 | 0.00130 | 0.00913 | 0.03912 | 0.47601 | 0.003 |
| 2. | 1 | 0.80 | 0.767 | 0.00061 | 0.00061 | 0.00428 | 0.01834 | 0.22312 | 0.002 |
| 3. | 2 | 91.40 | 0.471 | 0.04305 | 0.08610 | 0.60267 | 2.58288 | 31.42503 | 0.215 |
| 4. | 3 | 5.94 | 1.896 | 0.01127 | 0.03380 | 0.23663 | 1.01411 | 12.33838 | 0.085 |
| 5. | 3 | 20.72 | 1.908 | 0.03953 | 0.11858 | 0.83009 | 3.55752 | 43.28320 | 0.296 |

Table S6. Mercury content in nutricosmetics.

| **No** | **Max**  **daily dosage** | **Content of Hg [µg/kg]** | **Weight of 1 portion [g]** | **Intake of Hg [µg]** | | | | | **% PTWI** |
| --- | --- | --- | --- | --- | --- | --- | --- | --- | --- |
|  |  |  |  | **One portion** | **Daily (EDI)** | **Weekly (EWI)** | **Monthly** | **Annual** |  |
| 1. | 1 | 1.20 | 0.587 | 0.00070 | 0.00070 | 0.00492 | 0.02110 | 0.25668 | 0.002 |
| 2. | 1 | 0.56 | 0.801 | 0.00044 | 0.00044 | 0.00311 | 0.01334 | 0.16226 | 0.001 |
| 3. | 1 | 0.94 | 1.147 | 0.00108 | 0.00108 | 0.00757 | 0.03245 | 0.39479 | 0.003 |
| 4. | 1 | 2.93 | 1.608 | 0.00471 | 0.00471 | 0.03298 | 0.14134 | 1.71968 | 0.012 |
| 5. | 1 | 3.67 | 0.965 | 0.00354 | 0.00354 | 0.02479 | 0.10625 | 1.29267 | 0.009 |
| 6. | 1 | 1.13 | 0.533 | 0.00060 | 0.00060 | 0.00422 | 0.01810 | 0.22022 | 0.002 |
| 7. | 1 | 3.30 | 0.160 | 0.00053 | 0.00053 | 0.00369 | 0.01584 | 0.19266 | 0.001 |
| 8. | 2 | 4.12 | 0.507 | 0.00209 | 0.00418 | 0.02923 | 0.12527 | 1.52411 | 0.010 |
| 9. | 1 | 2.00 | 0.373 | 0.00074 | 0.00074 | 0.00521 | 0.02235 | 0.27188 | 0.002 |
| 10. | 15 | 22.00 | 0.205 | 0.00451 | 0.06765 | 0.47357 | 2.02959 | 24.69337 | 0.169 |
| 11. | 1 | 4.78 | 1.544 | 0.00738 | 0.00738 | 0.05168 | 0.22150 | 2.69494 | 0.018 |
| 12. | 1 | 2.48 | 0.592 | 0.00147 | 0.00147 | 0.01026 | 0.04397 | 0.53501 | 0.004 |
| 13. | 6 | 1.88 | 0.705 | 0.00133 | 0.00796 | 0.05570 | 0.23870 | 2.90417 | 0.020 |
| 14. | 2 | 2.15 | 0.351 | 0.00075 | 0.00151 | 0.01055 | 0.04522 | 0.55013 | 0.004 |
| 15. | 1 | 3.72 | 0.485 | 0.00180 | 0.00180 | 0.01263 | 0.05414 | 0.65871 | 0.005 |
| 16. | 1 | 1.11 | 0.511 | 0.00056 | 0.00056 | 0.00395 | 0.01694 | 0.20610 | 0.001 |
| 17. | 1 | 12.16 | 1.144 | 0.01392 | 0.01392 | 0.09741 | 0.41747 | 5.07920 | 0.035 |

Table S7. Mercury content in dietary supplements supporting the immunity.

| **No** | **Max**  **daily dosage** | **Content of Hg [µg/kg]** | **Weight of 1 portion [g]** | **Intake of Hg [µg]** | | | | | **% PTWI** |
| --- | --- | --- | --- | --- | --- | --- | --- | --- | --- |
|  |  |  |  | **One portion** | **Daily (EDI)** | **Weekly (EWI)** | **Monthly** | **Annual** |  |
| 1. | 4 | 0.34 | 0.674 | 0.00023 | 0.00090 | 0.00632 | 0.02709 | 0.32965 | 0.002 |
| 2. | 2 | 0.53 | 10.000 | 0.00530 | 0.01060 | 0.07420 | 0.31800 | 3.86900 | 0.027 |
| 3. | 1 | 0.61 | 10.000 | 0.00610 | 0.00610 | 0.04270 | 0.18300 | 2.22650 | 0.015 |
| 4. | 2 | 0.66 | 2.500 | 0.00166 | 0.00331 | 0.02317 | 0.09930 | 1.20815 | 0.008 |
| 5. | 3 | 1.55 | 6.046 | 0.00936 | 0.02808 | 0.19654 | 0.84233 | 10.24833 | 0.070 |
| 6. | 3 | 3.43 | 4.537 | 0.01555 | 0.04664 | 0.32651 | 1.39935 | 17.02539 | 0.117 |
| 7. | 2 | 2.62 | 0.502 | 0.00132 | 0.00263 | 0.01843 | 0.07897 | 0.96086 | 0.007 |
| 8. | 1 | 2.72 | 0.469 | 0.00128 | 0.00128 | 0.00894 | 0.03830 | 0.46597 | 0.003 |
| 9. | 2 | 2.10 | 0.549 | 0.00115 | 0.00230 | 0.01612 | 0.06908 | 0.84041 | 0.006 |
| 10. | 2 | 5.05 | 0.199 | 0.00100 | 0.00201 | 0.01407 | 0.06030 | 0.73361 | 0.005 |
| 11. | 2 | 2.17 | 0.267 | 0.00058 | 0.00116 | 0.00812 | 0.03478 | 0.42315 | 0.003 |
| 12. | 1 | 1.69 | 0.706 | 0.00120 | 0.00120 | 0.00837 | 0.03586 | 0.43627 | 0.003 |
| 13. | 1 | 0.76 | 2.171 | 0.00166 | 0.00166 | 0.01160 | 0.04969 | 0.60461 | 0.004 |
| 14. | 1 | 1.31 | 0.551 | 0.00072 | 0.00072 | 0.00505 | 0.02165 | 0.26346 | 0.002 |
| 15. | 1 | 11.88 | 1.000 | 0.01188 | 0.01188 | 0.08313 | 0.35628 | 4.33474 | 0.030 |
| 16. | 2 | 2.68 | 0.210 | 0.00056 | 0.00113 | 0.00789 | 0.03379 | 0.41115 | 0.003 |
| 17. | 2 | 5.47 | 0.447 | 0.00245 | 0.00489 | 0.03424 | 0.14673 | 1.78524 | 0.012 |
| 18. | 3 | 1.50 | 7.029 | 0.01057 | 0.03171 | 0.22200 | 0.95145 | 11.57592 | 0.079 |

Table S8. Mercury content in dietary supplements supporting the memory.

| **No** | **Max**  **daily dosage** | **Content of Hg [µg/kg]** | **Weight of 1 portion [g]** | **Intake of Hg [µg]** | | | | | **% PTWI** |
| --- | --- | --- | --- | --- | --- | --- | --- | --- | --- |
|  |  |  |  | **One portion** | **Daily (EDI)** | **Weekly (EWI)** | **Monthly** | **Annual** |  |
| 1. | 1 | 0.80 | 0.629 | 0.00050 | 0.00050 | 0.00352 | 0.01510 | 0.18367 | 0.001 |
| 2. | 2 | 1.24 | 0.560 | 0.00069 | 0.00139 | 0.00971 | 0.04160 | 0.50609 | 0.003 |
| 3. | 1 | 0.66 | 0.682 | 0.00045 | 0.00045 | 0.00315 | 0.01348 | 0.16404 | 0.001 |
| 4. | 2 | 0.74 | 3.975 | 0.00295 | 0.00591 | 0.04135 | 0.17721 | 2.15600 | 0.015 |
| 5. | 1 | 1.42 | 2.426 | 0.00345 | 0.00345 | 0.02418 | 0.10364 | 1.26094 | 0.009 |
| 6. | 6 | 2.09 | 0.472 | 0.00099 | 0.00592 | 0.04141 | 0.17748 | 2.15936 | 0.015 |
| 7. | 1 | 1.39 | 0.290 | 0.00040 | 0.00040 | 0.00282 | 0.01208 | 0.14692 | 0.001 |
| 8. | 1 | 0.96 | 1.193 | 0.00115 | 0.00115 | 0.00802 | 0.03436 | 0.41803 | 0.003 |
| 9. | 1 | 3.79 | 0.661 | 0.00250 | 0.00250 | 0.01751 | 0.07506 | 0.91319 | 0.006 |

Table S9. Mercury content in dietary supplements supporting the nervous system.

| **No** | **Max**  **daily dosage** | **Content of Hg [µg/kg]** | **Weight of 1 portion [g]** | **Intake of Hg [µg]** | | | | | **% PTWI** |
| --- | --- | --- | --- | --- | --- | --- | --- | --- | --- |
|  |  |  |  | **One portion** | **Daily**  **(EDI)** | **Weekly**  **(EWI)** | **Monthly** | **Annual** |  |
| 1. | 2 | 1.72 | 0.590 | 0.00101 | 0.00203 | 0.01418 | 0.06078 | 0.73951 | 0.005 |
| 2. | 3 | 2.47 | 0.583 | 0.00144 | 0.00432 | 0.03025 | 0.12965 | 1.57745 | 0.011 |
| 3. | 2 | 0.80 | 0.961 | 0.00077 | 0.00155 | 0.01082 | 0.04636 | 0.56403 | 0.004 |
| 4. | 1 | 4.64 | 1.990 | 0.00924 | 0.00924 | 0.06465 | 0.27707 | 3.37099 | 0.023 |

Table S10. Mercury content in dietary supplements for sore throat.

| **No** | **Max**  **daily dosage** | **Content of Hg [µg/kg]** | **Weight of 1 portion [g]** | **Intake of Hg [µg]** | | | | | **% PTWI** |
| --- | --- | --- | --- | --- | --- | --- | --- | --- | --- |
|  |  |  |  | **One portion** | **Daily (EDI)** | **Weekly (EWI)** | **Monthly** | **Annual** |  |
| 1. | 6 | 1.67 | 1.493 | 0.00250 | 0.01498 | 0.10484 | 0.44933 | 5.46689 | 0.037 |
| 2. | 6 | 1.18 | 1.450 | 0.00171 | 0.01023 | 0.07162 | 0.30694 | 3.73439 | 0.026 |
| 3. | 5 | 0.27 | 1.896 | 0.00052 | 0.00260 | 0.01818 | 0.07793 | 0.94809 | 0.006 |
| 4. | 4 | 3.20 | 3.511 | 0.01124 | 0.04497 | 0.31478 | 1.34907 | 16.41364 | 0.112 |
| 5. | 1 | 1.63 | 3.661 | 0.00597 | 0.00597 | 0.04182 | 0.17924 | 2.18078 | 0.015 |
| 6. | 4 | 2.44 | 3.457 | 0.00844 | 0.03374 | 0.23618 | 1.01221 | 12.31522 | 0.084 |
| 7. | 6 | 1.06 | 1.545 | 0.00163 | 0.00979 | 0.06852 | 0.29367 | 3.57303 | 0.024 |
| 8. | 4 | 1.50 | 3.508 | 0.00525 | 0.02099 | 0.14694 | 0.62976 | 7.66203 | 0.052 |
| 9. | 5 | 1.25 | 0.499 | 0.00062 | 0.00311 | 0.02176 | 0.09326 | 1.13470 | 0.008 |
| 10. | 4 | 0.42 | 3.138 | 0.00132 | 0.00527 | 0.03690 | 0.15816 | 1.92422 | 0.013 |
| 11. | 8 | 2.31 | 2.720 | 0.00629 | 0.05033 | 0.35232 | 1.50993 | 18.37077 | 0.126 |

Table S11. Mercury content in dietary supplements for supporting the functioning of the urinary track.

| **No** | **Max**  **daily dosage** | **Content of Hg [µg/kg]** | **Weight of 1 portion [g]** | **Intake of Hg [µg]** | | | | | **% PTWI** |
| --- | --- | --- | --- | --- | --- | --- | --- | --- | --- |
|  |  |  |  | **One portion** | **Daily (EDI)** | **Weekly (EWI)** | **Monthly** | **Annual** |  |
| 1. | 1 | 2.17 | 0.544 | 0.00118 | 0.00118 | 0.00825 | 0.03535 | 0.43008 | 0.003 |
| 2. | 3 | 0.67 | 0.863 | 0.00058 | 0.00173 | 0.01212 | 0.05196 | 0.63219 | 0.004 |
| 3. | 1 | 1.28 | 0.553 | 0.00071 | 0.00071 | 0.00494 | 0.02117 | 0.25755 | 0.002 |
| 4. | 1 | 2.48 | 0.530 | 0.00131 | 0.00131 | 0.00918 | 0.03935 | 0.47879 | 0.003 |
| 5. | 1 | 10.68 | 0.516 | 0.00551 | 0.00551 | 0.03856 | 0.16526 | 2.01072 | 0.014 |
| 6. | 1 | 13.84 | 0.415 | 0.00574 | 0.00574 | 0.04021 | 0.17235 | 2.09687 | 0.014 |
| 7. | 1 | 1.81 | 0.727 | 0.00132 | 0.00132 | 0.00923 | 0.03954 | 0.48109 | 0.003 |
| 8. | 2 | 9.62 | 0.607 | 0.00584 | 0.01168 | 0.08178 | 0.35047 | 4.26405 | 0.029 |
| 9. | 1 | 1.67 | 0.387 | 0.00065 | 0.00065 | 0.00452 | 0.01938 | 0.23575 | 0.002 |
| 10. | 6 | 0.68 | 0.713 | 0.00049 | 0.00293 | 0.02048 | 0.08778 | 1.06805 | 0.007 |

Table S12. Mercury content in dietary supplements supporting veins.

| **No** | **Max**  **daily dosage** | **Content of Hg [µg/kg]** | **Weight of 1 portion [g]** | **Intake of Hg [µg]** | | | | | **% PTWI** |
| --- | --- | --- | --- | --- | --- | --- | --- | --- | --- |
|  |  |  |  | **One portion** | **Daily**  **(EDI)** | **Weekly (EWI)** | **Monthly** | **Annual** |  |
| 1. | 1 | 5.98 | 1.040 | 0.00622 | 0.00622 | 0.04356 | 0.18670 | 2.27153 | 0.016 |
| 2. | 1 | 1.27 | 0.544 | 0.00069 | 0.00069 | 0.00483 | 0.02069 | 0.25177 | 0.002 |
| 3. | 2 | 3.81 | 0.518 | 0.00197 | 0.00394 | 0.02761 | 0.11832 | 1.43958 | 0.010 |
| 4. | 1 | 3.72 | 0.507 | 0.00189 | 0.00189 | 0.01320 | 0.05657 | 0.68822 | 0.005 |
| 5. | 2 | 8.57 | 0.631 | 0.00541 | 0.01081 | 0.07570 | 0.32442 | 3.94714 | 0.027 |
| 6. | 2 | 3.65 | 0.832 | 0.00303 | 0.00607 | 0.04246 | 0.18196 | 2.21383 | 0.015 |

Table S13. Mercury content in dietary supplements supporting the vision.

| **No** | **Max**  **daily dosage** | **Content of Hg [µg/kg]** | **Weight of 1 portion [g]** | **Intake of Hg [µg]** | | | | | **% PTWI** |
| --- | --- | --- | --- | --- | --- | --- | --- | --- | --- |
|  |  |  |  | **One portion** | **Daily (EDI)** | **Weekly (EWI)** | **Monthly** | **Annual** |  |
| 1. | 1 | 1.27 | 0.422 | 0.00054 | 0.00054 | 0.00375 | 0.01608 | 0.19562 | 0.001 |
| 2. | 1 | 1.38 | 1.052 | 0.00145 | 0.00145 | 0.01015 | 0.04352 | 0.52951 | 0.004 |
| 3. | 1 | 4.46 | 0.871 | 0.00388 | 0.00388 | 0.02716 | 0.11641 | 1.41631 | 0.010 |
| 4. | 2 | 21.81 | 0.411 | 0.00896 | 0.01793 | 0.12550 | 0.53786 | 6.54395 | 0.045 |
| 5. | 1 | 1.39 | 0.615 | 0.00085 | 0.00085 | 0.00597 | 0.02557 | 0.31112 | 0.002 |

Table S14. Mercury content in dietary supplements supporting the vitality.

| **No** | **Max**  **daily dosage** | **Content of Hg [µg/kg]** | **Weight of 1 portion [g]** | **Intake of Hg [µg]** | | | | | **% PTWI** |
| --- | --- | --- | --- | --- | --- | --- | --- | --- | --- |
|  |  |  |  | **One portion** | **Daily (EDI)** | **Weekly**  **(EWI)** | **Monthly** | **Annual** |  |
| 1. | 1 | 0.76 | 0.617 | 0.00047 | 0.00047 | 0.00329 | 0.01410 | 0.17161 | 0.001 |
| 2. | 1 | 0.83 | 0.798 | 0.00066 | 0.00066 | 0.00464 | 0.01989 | 0.24205 | 0.002 |
| 3. | 1 | 1.11 | 0.472 | 0.00052 | 0.00052 | 0.00367 | 0.01573 | 0.19140 | 0.001 |
| 4. | 1 | 0.46 | 3.985 | 0.00182 | 0.00182 | 0.01275 | 0.05463 | 0.66472 | 0.005 |
| 5. | 1 | 1.83 | 0.732 | 0.00134 | 0.00134 | 0.00937 | 0.04016 | 0.48867 | 0.003 |
| 6. | 2 | 1.91 | 0.948 | 0.00181 | 0.00361 | 0.02530 | 0.10841 | 1.31903 | 0.009 |
| 7. | 1 | 1.53 | 1.443 | 0.00220 | 0.00220 | 0.01540 | 0.06602 | 0.80321 | 0.006 |
| 8. | 1 | 1.81 | 3.991 | 0.00721 | 0.00721 | 0.05045 | 0.21623 | 2.63083 | 0.018 |
| 9. | 1 | 2.65 | 0.300 | 0.00080 | 0.00080 | 0.00557 | 0.02388 | 0.29050 | 0.002 |
| 10. | 2 | 4.63 | 0.702 | 0.00325 | 0.00650 | 0.04550 | 0.19502 | 2.37269 | 0.016 |
| 11. | 1 | 5.32 | 1.226 | 0.00653 | 0.00653 | 0.04569 | 0.19582 | 2.38244 | 0.016 |
| 12. | 1 | 1.19 | 1.322 | 0.00157 | 0.00157 | 0.01102 | 0.04724 | 0.57469 | 0.004 |
| 13. | 3 | 0.71 | 0.309 | 0.00022 | 0.00065 | 0.00458 | 0.01963 | 0.23888 | 0.002 |
| 14. | 1 | 2.33 | 0.503 | 0.00117 | 0.00117 | 0.00821 | 0.03520 | 0.42833 | 0.003 |
| 15. | 2 | 1.63 | 0.537 | 0.00088 | 0.00175 | 0.01228 | 0.05265 | 0.64054 | 0.004 |
| 16. | 1 | 2.73 | 0.754 | 0.00205 | 0.00205 | 0.01438 | 0.06164 | 0.74995 | 0.005 |
| 17. | 4 | 3.66 | 31.214 | 0.11434 | 0.45735 | 3.20143 | 13.72043 | 166.93185 | 1.143 |

Table S15. Mercury content in dietary supplements with the vitamins and minerals.

| **No** | **Max**  **daily dosage** | **Content of Hg [µg/kg]** | **Weight of 1 portion [g]** | **Intake of Hg [µg]** | | | | | **% PTWI** |
| --- | --- | --- | --- | --- | --- | --- | --- | --- | --- |
|  |  |  |  | **One portion** | **Daily**  **(EDI)** | **Weekly (EWI)** | **Monthly** | **Annual** |  |
| 1. | 2 | 1.18 | 0.580 | 0.00068 | 0.00136 | 0.00955 | 0.04092 | 0.49792 | 0.003 |
| 2. | 1 | 1.74 | 0.505 | 0.00088 | 0.00088 | 0.00613 | 0.02629 | 0.31980 | 0.002 |
| 3. | 3 | 1.60 | 1.969 | 0.00314 | 0.00943 | 0.06603 | 0.28300 | 3.44322 | 0.024 |
| 4. | 4 | 1.36 | 5.685 | 0.00771 | 0.03086 | 0.21601 | 0.92575 | 11.26324 | 0.077 |
| 5. | 5 | 1.92 | 1.212 | 0.00233 | 0.01164 | 0.08145 | 0.34906 | 4.24685 | 0.029 |
| 6. | 4 | 4.20 | 0.754 | 0.00317 | 0.01266 | 0.08863 | 0.37984 | 4.62133 | 0.032 |
| 7. | 1 | 1.86 | 0.460 | 0.00086 | 0.00086 | 0.00599 | 0.02568 | 0.31246 | 0.002 |
| 8. | 1 | 2.09 | 1.098 | 0.00229 | 0.00229 | 0.01603 | 0.06871 | 0.83601 | 0.006 |
| 9. | 1 | 4.68 | 0.743 | 0.00348 | 0.00348 | 0.02433 | 0.10425 | 1.26838 | 0.009 |
| 10. | 1 | 1.18 | 1.424 | 0.00169 | 0.00169 | 0.01180 | 0.05058 | 0.61540 | 0.004 |
| 11. | 1 | 1.15 | 1.594 | 0.00183 | 0.00183 | 0.01283 | 0.05499 | 0.66908 | 0.005 |
| 12. | 1 | 1.63 | 1.148 | 0.00187 | 0.00187 | 0.01309 | 0.05610 | 0.68258 | 0.005 |
| 13. | 1 | 4.83 | 0.624 | 0.00302 | 0.00302 | 0.02111 | 0.09046 | 1.10054 | 0.008 |
| 14. | 2 | 1.39 | 1.088 | 0.00151 | 0.00302 | 0.02116 | 0.09067 | 1.10320 | 0.008 |
| 15. | 1 | 1.58 | 0.901 | 0.00142 | 0.00142 | 0.00995 | 0.04265 | 0.51895 | 0.004 |
| 16. | 1 | 0.78 | 1.861 | 0.00146 | 0.00146 | 0.01021 | 0.04377 | 0.53254 | 0.004 |
| 17. | 1 | 0.60 | 4.323 | 0.00258 | 0.00258 | 0.01804 | 0.07730 | 0.94043 | 0.006 |
| 18. | 2 | 2.77 | 0.698 | 0.00193 | 0.00387 | 0.02707 | 0.11601 | 1.41143 | 0.010 |
| 19. | 1 | 2.83 | 0.596 | 0.00169 | 0.00169 | 0.01182 | 0.05064 | 0.61607 | 0.004 |
| 20. | 1 | 3.53 | 1.102 | 0.00389 | 0.00389 | 0.02720 | 0.11657 | 1.41826 | 0.010 |
| 21. | 1 | 42.96 | 0.837 | 0.03596 | 0.03596 | 0.25170 | 1.07870 | 13.12419 | 0.090 |
| 22. | 1 | 7.83 | 1.643 | 0.01287 | 0.01287 | 0.09009 | 0.38609 | 4.69741 | 0.032 |
| 23. | 1 | 3.17 | 4.355 | 0.01380 | 0.01380 | 0.09661 | 0.41403 | 5.03736 | 0.035 |

Table S16. Mercury content in dietary supplements supporting the weight loss.

| **No** | **Max**  **daily dosage** | **Content of Hg [µg/kg]** | **Weight of 1 portion [g]** | **Intake of Hg [µg]** | | | | | **% PTWI** |
| --- | --- | --- | --- | --- | --- | --- | --- | --- | --- |
|  |  |  |  | **One portion** | **Daily (EDI)** | **Weekly (EWI)** | **Monthly** | **Annual** |  |
| 1. | 2 | 1.21 | 0.709 | 0.00086 | 0.00172 | 0.01203 | 0.05156 | 0.62729 | 0.004 |
| 2. | 2 | 1.01 | 1.138 | 0.00115 | 0.00231 | 0.01614 | 0.06917 | 0.84154 | 0.006 |
| 3. | 6 | 0.54 | 0.672 | 0.00036 | 0.00216 | 0.01510 | 0.06471 | 0.78735 | 0.005 |
| 4. | 2 | 1.32 | 1.026 | 0.00135 | 0.00270 | 0.01893 | 0.08114 | 0.98716 | 0.007 |
| 5. | 2 | 1.78 | 0.602 | 0.00107 | 0.00214 | 0.01497 | 0.06415 | 0.78048 | 0.005 |
| 6. | 2 | 1.95 | 3.217 | 0.00626 | 0.01251 | 0.08760 | 0.37542 | 4.56766 | 0.031 |
| 7. | 2 | 2.00 | 6.587 | 0.01317 | 0.02635 | 0.18444 | 0.79044 | 9.61702 | 0.066 |
| 8. | 2 | 1.44 | 0.657 | 0.00095 | 0.00189 | 0.01326 | 0.05684 | 0.69160 | 0.005 |
| 9. | 2 | 1.36 | 2.023 | 0.00275 | 0.00549 | 0.03843 | 0.16471 | 2.00400 | 0.014 |
| 10. | 2 | 1.00 | 0.723 | 0.00072 | 0.00145 | 0.01012 | 0.04338 | 0.52779 | 0.004 |
| 11. | 1 | 1.77 | 8.984 | 0.01587 | 0.01587 | 0.11106 | 0.47597 | 5.79100 | 0.040 |
| 12. | 6 | 1.06 | 0.205 | 0.00022 | 0.00130 | 0.00911 | 0.03904 | 0.47499 | 0.003 |
| 13. | 4 | 5.33 | 0.735 | 0.00392 | 0.01567 | 0.10967 | 0.47002 | 5.71855 | 0.039 |
| 14. | 2 | 1.35 | 0.694 | 0.00093 | 0.00187 | 0.01308 | 0.05605 | 0.68191 | 0.005 |
| 15. | 2 | 0.52 | 0.562 | 0.00029 | 0.00059 | 0.00411 | 0.01764 | 0.21457 | 0.001 |
| 16. | 1 | 1.14 | 10.000 | 0.01140 | 0.01140 | 0.07980 | 0.34200 | 4.16100 | 0.029 |
| 17. | 2 | 2.68 | 0.210 | 0.00056 | 0.00113 | 0.00789 | 0.03379 | 0.41115 | 0.003 |
| 18. | 1 | 2.88 | 6.076 | 0.01752 | 0.01752 | 0.12262 | 0.52551 | 6.39374 | 0.044 |
| 19. | 1 | 4.06 | 1.065 | 0.00432 | 0.00432 | 0.03025 | 0.12965 | 1.57745 | 0.011 |
| 20. | 2 | 17.26 | 0.181 | 0.00312 | 0.00625 | 0.04374 | 0.18747 | 2.28083 | 0.016 |
| 21. | 4 | 3.96 | 0.638 | 0.00253 | 0.01011 | 0.07076 | 0.30325 | 3.68959 | 0.025 |
| 22. | 1 | 6.81 | 1.048 | 0.00714 | 0.00714 | 0.04996 | 0.21411 | 2.60496 | 0.018 |
| 23. | 3 | 2.78 | 0.982 | 0.00273 | 0.00820 | 0.05741 | 0.24605 | 2.99361 | 0.021 |
| 24. | 2 | 2.15 | 1.470 | 0.00317 | 0.00633 | 0.04433 | 0.18998 | 2.31146 | 0.016 |
| 25. | 2 | 9.52 | 0.702 | 0.00668 | 0.01336 | 0.09352 | 0.40081 | 4.87657 | 0.033 |

Table S17. Mercury content in other, unclassified dietary supplements.

| **No** | **Max**  **daily dosage** | **Content of Hg [µg/kg]** | **Weight of 1 portion [g]** | **Intake of Hg [µg]** | | | | | **% PTWI** |
| --- | --- | --- | --- | --- | --- | --- | --- | --- | --- |
|  |  |  |  | **One portion** | **Daily (EDI)** | **Weekly (EWI)** | **Monthly** | **Annual** |  |
| 1. | 4 | 0.88 | 2.426 | 0.00213 | 0.00853 | 0.05971 | 0.25589 | 3.11338 | 0.021 |
| 2. | 1 | 1.36 | 1.227 | 0.00167 | 0.00167 | 0.01170 | 0.05014 | 0.60998 | 0.004 |
| 3. | 1 | 2.24 | 1.376 | 0.00308 | 0.00308 | 0.02157 | 0.09243 | 1.12452 | 0.008 |
| 4. | 1 | 2.35 | 0.545 | 0.00128 | 0.00128 | 0.00897 | 0.03844 | 0.46767 | 0.003 |
| 5. | 5 | 0.54 | 3.451 | 0.00187 | 0.00935 | 0.06547 | 0.28057 | 3.41356 | 0.023 |
| 6. | 2 | 1.10 | 3.972 | 0.00439 | 0.00877 | 0.06139 | 0.26311 | 3.20111 | 0.022 |
| 7. | 1 | 1.69 | 0.113 | 0.00019 | 0.00019 | 0.00134 | 0.00572 | 0.06962 | <0.001 |
| 8. | 3 | 1.08 | 0.640 | 0.00069 | 0.00207 | 0.01447 | 0.06204 | 0.75476 | 0.005 |
| 9. | 3 | 1.13 | 1.018 | 0.00115 | 0.00345 | 0.02414 | 0.10344 | 1.25851 | 0.009 |
| 10. | 1 | 2.47 | 0.630 | 0.00156 | 0.00156 | 0.01089 | 0.04666 | 0.56775 | 0.004 |
| 11. | 2 | 2.34 | 4.990 | 0.01166 | 0.02331 | 0.16319 | 0.69940 | 8.50935 | 0.058 |
